# Supplementary material for: Identification of photoperiod-induced specific miRNAs in the adrenal glands of Sunite sheep (Ovis aries)
Source: Front Vet Sci. 2022 Jul 22;9:888207. doi: 10.3389/fvets.2022.888207 (PMC9354845; doi:10.3389/fvets.2022.888207)
Supplement: Supplementary file 1 [file Data_Sheet_1.ZIP › Supplementary file/miRDeep2 file.zip!/miRDeep2 file/Know_Structure/SPLP42A2/oar-mir-377.pdf]

miRBase precursor : oar-mir-377
Total read count : 54
oar-miR-377-5p read count : 17
oar-miR-377-3p read count : 28
remaining reads : 9

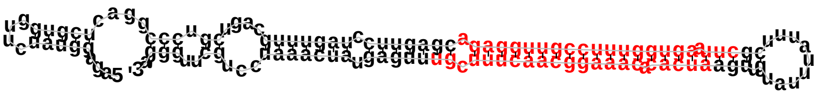

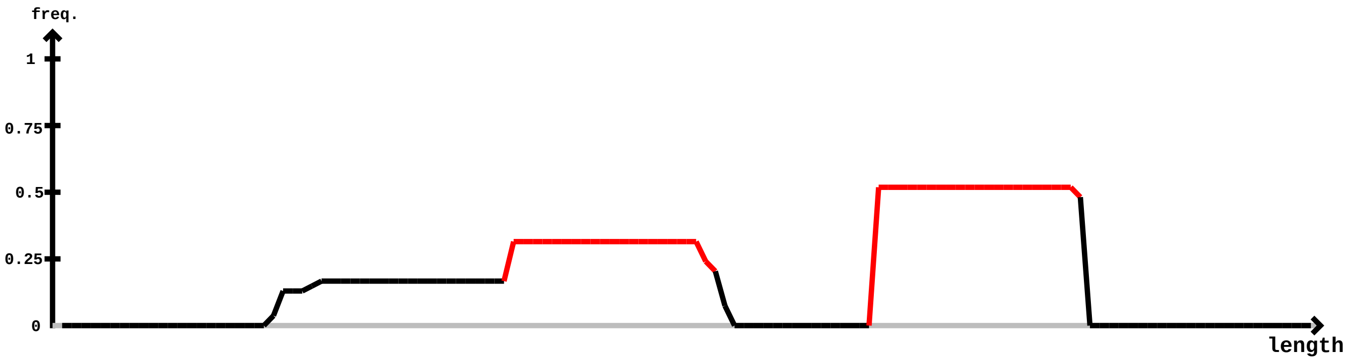


```
                                                                                  oar-miR-377-3p
                                                  oar-miR-377-5p
5'- agagguaucuuggugcucaggcccugcugacguuugauccuugagcagagguugccuuuggugaauucgcuuuauuuauguugaaucacacaaaggcaacuuucguuugaguaucaaauccugcuuggga   -3'    exp
    ...(((((....)))))....(((.((....(((((((.(((((((.((((((((((((((((.((((((..........).)))))))).))))))))))))).))))))).)))))))...))..))).    reads      mm
    .....................cccugcugacguuugauccuugagc....................................................................................    2          0
    ......................ccugcugacguuugauccuugagc....................................................................................    5          0
    .........................gcugacguuugauccuugagc....................................................................................    1          0
    ..........................cugacguuugauccuugagc....................................................................................    1          0
    ..............................................agagguugccuuuggugaau................................................................    4          0
    ..............................................agagguugccuuuggugaauu...............................................................    2          0
    ..............................................agagguugccuuuggugaauuc..............................................................    7          0
    ..............................................agagguugccuuuggugaauucg.............................................................    4          0
    ....................................................................................aucacacaaaggcaacuuucg.........................    2          0
    ....................................................................................aucacacaaaggcaacuuucgu........................    26         0
```
